# Additions to the lists of publications of Terry Erwin, taxa described by him and named after him after 2015

**DOI:** 10.3897/zookeys.1044.69603

**Published:** 2021-06-16

**Authors:** Lyubomir Penev, Achille Casale, Yordanka Banalieva

**Affiliations:** 1 Institute of Biodiversity & Ecosystem Research – Bulgarian Academy of Sciences, Sofia, Bulgaria; 2 Pensoft Publishers, Sofia, Bulgaria; 3 Corso Raffaello 12, 10126 Torino, Italy

**Keywords:** Biodiversity, bibliography, Carabidae, Coleoptera, Hemiptera, Hymenoptera, Insecta, patronyms

## Abstract

A paper on the occasion of the 75^th^ birthday of Terry Lee Erwin (1940–2020), an outstanding biologist and founding Editor-in-Chief of ZooKeys, was published in 2015 and contained complete lists of Erwin’s publications, patronyms (taxa named after him) and new taxa published by him. The present paper aims to complement these lists with all new information published after 2015, including the papers in the present special issue of ZooKeys dedicated to the blessed memory of Terry Lee Erwin.

## Introduction

The ZooKeys founding Editor-in-Chief and world-known entomologist Terry Lee Erwin passed away on 11^th^ of May 2020, leaving a sad feeling of emptiness and loss in our hearts! In recognition of his outstanding scientific achievements and dedication to ZooKeys from the very start of the journal, Pensoft and Terry’s colleagues and friends organised a memorial volume in ZooKeys published today and of which the present article is a part. It encompasses the list of 10 publications of Terry Erwin, 31 new taxa described by him and 16 taxa named after him, published after the Festschrift paper on the occasion of Terry’s 75^th^ birthday (ZooKeys Editorial Office 2015), including also the patronyms described to honor Terry in the present memorial volume. In total, this makes 268 publications, 67 patronyms and the impressive 472 taxa described as new to science by Terry Erwin.

## Additions to the List of publications of Terry Erwin published after 2015

For the list of publications before 2015 see ZooKeys Editorial Office (2015), https://doi.org/10.3897/zookeys.541.7316.

Erwin TL, Kavanaugh DH, Maddison DR (2020) After 157 years, a second specimen and species of the phylogenetically enigmatic and previously monobasic genus *Nototylus* Gemminger & Harold, 1868 (Coleoptera, Carabidae, Nototylini). ZooKeys 927: 65–74. https://doi.org/10.3897/zookeys.927.49584

Maddison DR, Kanda K, Boyd OF, Faille A, Porch N, Erwin TL, Roig-Juñent S (2019) Phylogeny of the beetle supertribe Trechitae (Coleoptera: Carabidae): unexpected clades, isolated lineages, and morphological convergence. Molecular Phylogenetics and Evolution 132: 151–176. https://doi.org/10.1016/j.ympev.2018.11.006

Erwin TL, Aldebron C (2018) Neotropical *Thoasia* Liebke, 1939 and *Straneotia* Mateu, 1961 of the Cryptobatida group, subtribe Agrina: Taxonomic revisions with notes on their ways of life (Insecta, Coleoptera, Carabidae, Lebiini). ZooKeys 742: 57–90. https://doi.org/10.3897/zookeys.742.22900

Erwin TL, Henry SC (2017) *Hyboptera* Chaudoir, 1872 of the Cryptobatida group of subtribe Agrina: A taxonomic revision with notes on their ways of life (Insecta, Coleoptera, Carabidae, Lebiini). ZooKeys 714: 61–127. https://doi.org/10.3897/zookeys.714.15113

Erwin TL, Zamorano LS, Geraci CJ (2017) Amazonian rainforests and their richness of Coleoptera, a dominant life form in the *Critical Zone* of the Neotropics. In: Foottit R, Adler P (Eds) Insect Biodiversity: Science and Society, 2^nd^ edn. Blackwell Publishing, New Jersey. https://doi.org/10.1002/9781118945568.ch4

Boyd OF, Erwin TL (2016) Taxonomic review of New World Tachyina (Coleoptera, Carabidae): descriptions of new genera, subgenera, and species, with an updated key to the subtribe in the Americas. ZooKeys 626: 87–123. https://doi.org/10.3897/zookeys.626.10033

García-Robledo C, Kuprewicz PK, Staines CL, Erwin TL, Kress WJ (2016) Limited tolerance by insects to high temperatures across tropical elevation gradients and the implications of global warming for extinction. Proceedings of the National Academy of Sciences of the United States of America 113(3): 680–685. https://doi.org/10.1073/pnas.1507681113

Levine NM, Zhang K, Longo M, Baccini A, Phillips OL, Lewis SL, Alvarez, E, de Andrade ACS, Brienen R, Erwin T, Feldpausch TR, Mendoza ALM, Vargas PN, Prieto A, Espejo JES, Malhi Y, Moorcroft PR (2016) Ecosystem heterogeneity determines the resilience of the Amazon to climate change. Proceedings of the National Academy of Sciences of the United States of America 113(3): 793–797. https://doi.org/10.1073/pnas.1511344112

Ashworth AC, Erwin TL (2016) *Antarctotrechus balli* sp. n. (Carabidae, Trechini): the first ground beetle from Antarctica. ZooKeys 635: 109–122. https://doi.org/10.3897/zookeys.635.10535

Erwin TL, Micheli C, Chaboo CS (2015) Beetles (Coleoptera) of Peru: A Survey of the Families. Carabidae. Journal of the Kansas Entomological Society 88(2): 151–162. https://doi.org/10.2317/kent-88-02-151-162.1

**Table 1.** Additions to the List of taxa named after Terry Erwin, published after 2015. See table 1 in: ZooKeys Editorial Office (2015), https://doi.org/10.3897/zookeys.541.7316.

Order	Family	Genus	Species	Taxon author	Publication date	Reference	Locality
Coleoptera	Carabidae	* Erwinanillus *		Giachino, Eberhard & Perina	2021: 275	Giachino et al. (2021)	WA, Southern Goldfields region
Coleoptera	Carabidae	* Paratachys *	* terryli *	Liebherr	2021: 235	Liebherr (2021)	USA: Hawaii
Coleoptera	Carabidae	* Diplocheila *	* erwini *	Allegro & Giachino	2021: 438	Allegro and Giachino (2021)	Cambodia
Coleoptera	Carabidae	subgenus *Erwinebria*		Kavanaugh	2021: 120	Kavanaugh et al. (2021)	North America
Coleoptera	Curculionidae	* Coptoborus *	* erwini *	Smith & Cognato	2021: 651	Smith and Cognato (2021)	Ecuador (Orellana)
Coleoptera	Carabidae	* Tasmanitachoides *	* erwini *	Maddison & Porch	2021: 186	Maddison and Porch (2021)	Australia: Tasmania
Coleoptera	Carabidae	* Yalongaphaenops *	* erwini *	Belousov & Kabak	2021: 207	Belousov and Kabak (2021)	China, Sichuan Province,
Coleoptera	Chrysomelidae	* Hemilactica *	* erwini *	Konstantinov	2021: 591	Konstantinov (2021)	Dominican Republic
Coleoptera	Curculionidae	* Conotrachelus *	* terryerwini *	Anderson	2021: 723	Anderson (2021)	Costa Rica
Coleoptera	Carabidae	Bembidion (Ocydromus)	* terryerwini *	Neri & Toledano	2021: 222	Neri and Toledano (2021)	Iran
Coleoptera	Carabidae	* Coptocarpus *	* erwini *	Will & Guéorguiev	2021: 388	Will and Guéorguiev (2021)	New Caledonia
Coleoptera	Carabidae	* Calleida *	* erwini *	Casale	2021: 497	Casale (2021)	Peru, Madre de Dios
Hymenoptera	Braconidae	Aleiodes	* terryerwini *	Sharkey	2021: 582	Sharkey et al. (2021)	Costa Rica
Coleoptera	Histeridae	Phelister	* erwini *	Caterino & Tishechkin	2020: 25	Caterino and Tishechkin (2020)	Ecuador, Brasil, Perú, Colombia, Panama
Hemiptera	Membracidae	* Cladonota *	* erwini *	Flynn	2019: 413	Flynn (2019)	Ecuador
Hymenoptera	Braconidae	* Zelomoprpha *	* terryerwini *	Meierotto	2019: 134	Meierotto et al. (2019)	Costa Rica

**Table 2.** Additions to the List of genera and subgenera described by Terry Erwin after 2015. See table 3 in: ZooKeys Editorial Office (2015), https://doi.org/10.3897/zookeys.541.7316).

Genus/Subgenus	Type species	Genus author	Publication date	Reference	Locality
* Antarctotrechus *	*Antarctotrechus balli* Ashworth & Erwin	Ashworth & Erwin	2016: 114	Ashworth and Erwin (2016)	Antarctica, Oliver Bluffs
* Tachyxysta *	*Tachyxysta howdenorum* Boyd & Erwin	Boyd & Erwin	2016: 97	Boyd and Erwin (2016)	Mexico, Honduras
* Stigmatachys *	*Stigmatachys uvea* Boyd & Erwin	Boyd & Erwin	2016: 111	Boyd and Erwin (2016)	Perú
* Nothoderis *	*Tachys rufotestacea* Hayward	Boyd & Erwin	2016: 113	Boyd and Erwin (2016)	North, Central and Amazonian South America
Scolistichus (subgenus of Meotachys)	*Meotachys riparius* Boyd & Erwin	Boyd & Erwin	2016: 114	Boyd and Erwin (2016)	Amazon basin (Ecuador, Brazil, Perú, Colombia)
Hylotachys (subgenus of Meotachys)	*Meotachys ballorum* Boyd & Erwin	Boyd & Erwin	2016: 115	Boyd and Erwin (2016)	Amazon basin (Ecuador, Brazil, Perú)
Ammotachys (subgenus of Elaphropus)	*Elaphropus marchantarius* Boyd & Erwin	Boyd & Erwin	2016: 102	Boyd and Erwin (2016)	Amazon basin (Colombia, Brasil, Venezuela)
Idiotachys (subgenus of Elaphropus)	*Elaphropus acutifrons* Boyd & Erwin	Boyd & Erwin	2016: 104	Boyd and Erwin (2016)	Brazil

**Table 3.** Additions to the List of species described by Terry Erwin after 2015. See table 4 in: ZooKeys Editorial Office (2015), https://doi.org/10.3897/zookeys.541.7316.

Genus	Species	Author	Publication date	Reference	Locality
* Nototylus *	* balli *	Erwin & Kavanaugh	2020: 69	Erwin, Kavanaugh and Maddison (2020)	French Guiana
* Thoasia *	* surinamensis *	Erwin & Aldebron	2018: 68	Erwin and Aldebron (2018)	Suriname
* Thoasia *	* pterosmaragdos *	Aldebron & Erwin	2018: 69	Erwin and Aldebron (2018)	French Guiana
* Thoasia *	* manu *	Erwin & Aldebron	2018: 70	Erwin and Aldebron (2018)	Ecuador, Perú
* Straneotia *	* confundis *	Aldebron & Erwin	2018: 79	Erwin and Aldebron (2018)	Ecuador
* Straneotia *	* moi *	Aldebron & Erwin	2018: 83	Erwin and Aldebron (2018)	French Guiana
* Straneotia *	* cylindroceps *	Erwin & Aldebron	2018: 85	Erwin and Aldebron (2018)	French Guiana
* Hyboptera *	* biolat *	Erwin & Henry	2017: 82	Erwin and Henry (2017)	Perú
* Hyboptera *	* vestiverdis *	Henry & Erwin	2017: 83	Erwin and Henry (2017)	Perú
* Hyboptera *	* scheelea *	Erwin & Henry	2017: 87	Erwin and Henry (2017)	Perú
* Hyboptera *	* shasta *	Erwin	2017: 88	Erwin and Henry (2017)	Brazil
* Hyboptera *	* tiputini *	Erwin & Henry	2017: 91	Erwin and Henry (2017)	Ecuador
* Hyboptera *	* tepui *	Erwin & Henry	2017: 92	Erwin and Henry (2017)	Venezuela
* Hyboptera *	* lucida *	Henry & Erwin	2017: 108	Erwin and Henry (2017)	French Guiana
* Antarctotrechus *	* balli *	Ashworth & Erwin	2016: 115	Ashworth and Erwin (2016)	Antarctica, Oliver Bluffs
* Tachyxysta *	* howdenorum *	Boyd & Erwin	2016: 98	Boyd and Erwin (2016)	Mexico, Honduras
* Stigmatachys *	* uvea *	Boyd & Erwin	2016: 112	Boyd and Erwin (2016)	Perú
Meotachys (Scolistichus)	* riparius *	Boyd & Erwin	2016: 115	Boyd and Erwin (2016)	Colombia
Meotachys (Hylotachys)	* ballorum *	Boyd & Erwin	2016: 116	Boyd and Erwin (2016)	(Ecuador, Brazil, Colombia)
Meotachys (Hylotachys)	* rubrum *	Boyd & Erwin	2016: 117	Boyd and Erwin (2016)	Perú
Elaphropus (Ammotachys)	* marchantarius *	Boyd & Erwin	2016: 103	Boyd and Erwin (2016)	Amazon basin (Colombia, Brazil, Venezuela)
Elaphropus (Idiotachys)	* acutifrons *	Boyd & Erwin	2016: 106	Boyd and Erwin (2016)	Brazil
Elaphropus (Nototachts)	* occidentalis *	Boyd & Erwin	2016: 107	Boyd and Erwin (2016)	Perú, Brazil, Argentina
